# Concept Review of a Cloud-Based Smart Battery Management System for Lithium-Ion Batteries: Feasibility, Logistics, and Functionality

**DOI:** 10.3390/batteries8020019

**Published:** 2022-02-18

**Authors:** Manh-Kien Tran, Satyam Panchal, Tran Dinh Khang, Kirti Panchal, Roydon Fraser, Michael Fowler

**Affiliations:** 1 Department of Chemical Engineering, University of Waterloo, 200 University Avenue West, Waterloo, ON N2L3G1, Canada; mfowler@uwaterloo.ca; 2 Department of Mechanical and Mechatronics Engineering, University of Waterloo, 200 University Avenue West, Waterloo, ON N2L3G1, Canada; satyam.panchal@uwaterloo.ca (S.P.); rafraser@uwaterloo.ca (R.F.); 3 Department of Information Systems, Hanoi University of Science and Technology, Hanoi 10000, Vietnam; khangtd@soict.hust.edu.vn; 4 Department of Mathematics, Bhailalbhai & Bhikhabhai Institute of Technology (BBIT), Vallabh Vidyanagar 388120, Gujarat, India; kjpanchal@bbit.ac.in

**Keywords:** lithium-ion battery, battery modeling, battery management system, smart systems, cloud infrastructure, Internet of things

## Abstract

Energy storage plays an important role in the adoption of renewable energy to help solve climate change problems. Lithium-ion batteries (LIBs) are an excellent solution for energy storage due to their properties. In order to ensure the safety and efficient operation of LIB systems, battery management systems (BMSs) are required. The current design and functionality of BMSs suffer from a few critical drawbacks including low computational capability and limited data storage. Recently, there has been some effort in researching and developing smart BMSs utilizing the cloud platform. A cloud-based BMS would be able to solve the problems of computational capability and data storage in the current BMSs. It would also lead to more accurate and reliable battery algorithms and allow the development of other complex BMS functions. This study reviews the concept and design of cloud-based smart BMSs and provides some perspectives on their functionality and usability as well as their benefits for future battery applications. The potential division between the local and cloud functions of smart BMSs is also discussed. Cloud-based smart BMSs are expected to improve the reliability and overall performance of LIB systems, contributing to the mass adoption of renewable energy.

## 1. Introduction

Since the energy demand is projected to increase significantly in the future, the need for renewable energy is as high as ever nowadays. With that, the role of energy storage in the power grid is becoming more and more important [1,2]. As an enabling technology, it can help integrate more renewable energy sources such as solar and wind into the grid, lowering electricity costs and reducing environmental impact [3,4,5]. Lithium-ion batteries (LIBs) have recently gained increasing interest as excellent energy storage systems (ESSs) due to their high energy and power density, long lifespan, and low self-discharge [6,7]. In recent years, over 90% of large-scale energy storage capacity was provided by LIBs annually in the United States [8]. LIBs are used in many real-world applications, such as electric vehicles (EVs), battery energy storage systems (BESSs), and small portable devices such as laptops and smartphones [9]. Due to the increasing interest in LIBs, researchers have been focusing on increasing the energy density and lowering the costs of LIBs, allowing the battery technology to advance further [10,11,12].

In most real-world applications, the battery management system (BMS) is a mandatory component, serving the purpose of monitoring the battery’s health and safety. The role of the BMS becomes more significant in applications that have a large number of battery cells such as electric vehicles and battery storage power stations [13,14]. The BMS is a battery monitoring device, combining electronic hardware and software, that monitors and takes actions to protect the battery from certain usage or other conditions that could damage or shorten the life of the cells [15,16]. Originally, the main functions of the BMS were battery safety and protection by setting operational voltage, current, and temperature thresholds, as well as cell balancing. Over the years, research has been conducted on BMSs, resulting in further developments in improvement in performance, functionalities, and capabilities. Currently, there are more functions in BMSs that help the battery perform better and safer, including cell monitoring, battery safety and protection, state of charge (SOC) estimation, state of health (SOH) estimation, cell balancing, thermal management, and charging control [17,18]. Since there are concerns regarding the safety, reliability, and performance of LIBs, especially in standalone systems, a well-designed battery management system (BMS) is critical. In addition, battery models are used in the BMS to predict working voltage, power, and energy capability, estimate SOC and SOH, detect faults, and control battery operation [19]. They have a significant role in ensuring reliable performance and safety, improving the usage efficiency of the batteries, and avoiding malfunctions and catastrophic failures. It is important to have accurate and reliable battery models in the BMS.

Despite the recent works in developing better BMSs, there are still many downsides to the current design. For example, the current infrastructure of the BMS only allows it to utilize simple battery models like the equivalent circuit models or simplified electrochemical models, since it is limited in computing capability and data storage. There have been other proposed algorithms, using data-driven and machine learning techniques with more detailed electrical electrochemical models, but they cannot be implemented in the current BMS design due to the limited local computing resources [20,21]. Another disadvantage of the current BMS design is the lack of flexibility in its usage, as its algorithms are often hard programmed into the BMS, and changing the firmware requires a great effort. The current BMS also does not allow for a plug-and-go style and needs to be programmed specifically for different battery applications. These negative aspects of the BMS are mainly caused by the locality of the applications, and can be eliminated by a software and hardware infrastructure of the BMS that allows for the use of the Internet of things (IoT) and cloud applications. Recently, there has been some initial research into using IoT and cloud computing and storage in BMS applications [22,23,24]. However, the concept of cloud-based BMS has not been properly reviewed and introduced. In this study, the concept of a cloud-based smart BMS, utilizing the advantages of cloud computing and cloud storage, is reviewed. This study also provides some perspectives on the potential design, usage, benefits, and drawbacks of the cloud-based smart BMS for future battery applications. As well, there are discussions about the potential division between the local and cloud functions of the smart BMS. The rest of the study is as follows. Section 2 describes the current BMS application in more detail while Section 3 outlines the cloud-based smart BMS concept and design. Section 4 discusses the potential functionalities and capabilities of the smart BMS, as well as its integration approach and potential risks. Section 5 gives some concluding remarks about the concept of cloud-based BMSs.

## 2. Review of the Current Battery Management System (BMS)

Usually, the BMS for Li-ion battery systems has different layers for cells, modules, and packs. There is the “master” unit which is the main control and computing device, and there are many “slave” units that are used to monitor the voltage, current, and temperature of individual cells [25,26,27]. The “master” and “slave” units are constantly in communication with each other. The “slave” units monitor the cells and modules and collect data through sensors, then send the data to the “master” unit for processing and further control actions. The BMS also communicates with the contactors, in order to assure safety and also to regulate the input and output current for the battery system. The “master” unit can also be in communication with a user interface for applications that require more involved control and monitoring. The current BMS design is offline and local, meaning that it is operating without connection to the Internet or any servers. The data are usually stored locally in the “master” unit, or sometimes a separate unit if the data are exchanged asynchronously, and old data are deleted regularly to make room for newer data. The firmware is also hard programmed into the BMS, which means that the BMS has to be application specific and cannot be a one-size-fits-all solution.

The original two main functions of the BMS were monitoring and protecting the battery [28]. The monitoring function refers to the measurement of current, voltage, and temperature of the battery while the protecting function is responsible for bringing the system to a safe state when the measured values fall above or below their safe operational ranges [29,30]. The current and more modern BMS is relatively more complex and includes functions such as cell monitoring, cell balancing, battery safety and protection, state estimation, and thermal management [27,28,31]:

- Cell monitoring: This function involves the acquisition of the current, voltage, and temperature of each cell in the system. The measurements need to reach a certain degree of accuracy, as the data are often used to perform other functions in the BMS. Moreover, the BMS is also responsible for communication.
- Cell balancing: The imbalance of cell capacity can be caused by inconsistency in capacity and material quality during operation. Cell balancing is to redistribute the energy and maintain all cells at a similar SOC level. There are two cell balancing approaches: active and passive balancing. Active balancing is to transfer excess energy in higher SOC cells into lower SOC cells until they reach the same level. Passive balancing dissipates excess energy of higher SOC cells directly into heat to be removed. The function of cell balancing is necessary because battery capacity and lifetime will be reduced without it.
- Battery safety and protection: A main function of the BMS is to ensure the safety of the battery and protect it from operating at conditions that are harmful to both the battery and the users. Hazardous conditions are sometimes caused by the chemical characteristics of the battery. Fault diagnosis is a significant function of the BMS to ensure safe operation. Fault diagnosis algorithms detect the fault, identify its location and type, and perform necessary actions to reduce the effect of the fault. Fault diagnosis methods can be categorized into model-based, knowledge-based, and data-driven methods. The BMS also sets safety limits to protect the battery from working beyond the safe operating range of current, voltage, and temperature.
- State estimation: This mainly refers to the estimation of SOC and SOH. An accurate estimation of the battery SOC is necessary because it enables long battery life, prevention from battery failure, efficient operation, and accurate calculations of SOH and cell balancing. SOC estimation methods can be classified into the look-up table method, the coulomb counting method, model-based estimation methods, data-driven estimation methods, and the hybrid method. SOH estimation is crucial in selected energy management strategies to prolong battery life and appropriately arrange for the replacement of the battery. SOH can be estimated by direct measurement methods, indirect analysis methods, adaptive algorithms, and data-driven methods. Despite the importance of state estimation, the SOC and SOH values cannot be measured directly from the battery. Therefore, algorithms need to be developed to estimate the SOC and SOH of the battery based on the measurable data.
- Thermal management: The performance of the battery is usually affected by its temperature due to the effect of temperature on degradation and internal resistance. The battery thermal management system (BTMS) can help decrease maximum battery temperature and temperature differences inside the pack. There are three classes of BTMS, including active, passive, and hybrid. Active BTMS are air-based, liquid-based, and thermoelectric, whereas passive BTMS are phase change material (PCM)-based and heat-pipe-based. Hybrid BTMS use combinations of active and passive approaches such as PCM with air circulation, PCM with liquid circulation, and PCM with heat pipe. Without proper thermal management, the battery pack is susceptible to thermal runaway propagation as overheating is a direct trigger of thermal runaway.

**Figure 1:**
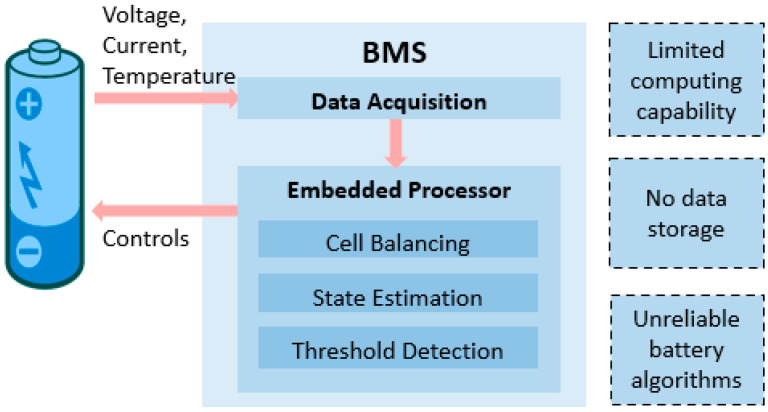
Current battery management system (BMS) design and functions.

Figure 1 shows the current BMS design and some of its limitations. A major issue with the offline BMS is the unreliability of state estimation algorithms [32]. Various model-based condition monitoring algorithms have been proposed to estimate battery states, including the Kalman filter [33,34] and the dual sliding mode observer [35]. Some commonly used battery models in the BMS are electrical models [36,37], electrochemical models [38], thermal models [39], and coupled models [40]. However, due to the complex internal principles and uncertain working conditions, it is difficult to establish a battery model that can represent the battery’s dynamic characteristics accurately [41]. Therefore, more complex data-driven algorithms are required to accurately estimate SOC, SOH, and RUL throughout the entire lifespan of the battery [42,43,44]. However, despite the achievable development of these algorithms, they cannot be integrated into the BMS due to its limited computing capability and data storage [45,46].

Another major issue in the current BMS is the lack of reliable real-time fault diagnosis algorithms [31,47]. Some battery faults include sensor faults, cell connection defects, internal short-circuiting, external short-circuiting, overheating, and thermal runaway [48,49]. These faults can cause major performance and safety issues, and hence they need to be identified as early as possible [50]. Various model-based methods currently used in the BMS utilize battery models and algorithms such as sliding mode observer [51], adaptive unscented Kalman filter [52], and structural analysis and sequential residual generator [53] to estimate parameters or residuals to detect battery faults. However, using these methods, many fault features are not reflected in the early stage of system failure. Some data-driven methods using signal processing [54,55] and machine learning [56,57,58] have been proposed to enable early and reliable fault detection but at the expense of high computing effort. Due to the data computing and storage limitations of the BMS, more reliable and accurate real-time fault diagnosis algorithms cannot be implemented.

## 3. Concept of the Cloud-Based Smart BMS

The limitations of the current BMS design have hindered the integration of large-scale LIB systems, thus slowing down the wide adoption of renewable energy [59,60]. The main reason for these issues is the computational capability and data storage constraints, as the BMS is currently designed to be locally integrated into the battery system [27]. Battery algorithms have been researched and developed while considering these constraints, and hence despite significant research efforts to improve these algorithms, they are not able to be practically implemented to improve the real-life performance of the BMS due to their higher complexity. The trade-off between accuracy and complexity has always been a problem for BMS developers. However, with the recently proposed design of a cloud-based smart BMS utilizing the advantages of cloud computing and cloud storage, this problem can potentially be resolved [22,23,24].

Kim et al. [22] proposed a novel battery monitoring and fault diagnosis approach using the cloud platform for large-scale lithium-ion BESSs. The proposed approach utilized a cyber-physical platform consisting of IoT components and cloud infrastructure. Battery algorithms for condition monitoring and fault diagnosis were built in the cloud-based BMS and then validated using a cyber-physical testbed and computational cost analysis. Li et al. [23] presented a cloud-based BMS, using IoT components to measure and transmit all relevant battery data to the cloud smoothly. The data were further used to build a digital twin for the battery system, where battery diagnostic algorithms evaluate the data to give a better understanding of the battery’s charge and aging level. A state of charge estimation method using an adaptive extended H-infinity filter and a state of health method using particle swarm optimization were also developed, both suiting the application of cloud-based BMS. The hardware and software of the proposed BMS were validated with prototypes in various experiments as well as under field operation for both stationary and mobile applications. Yang et al. [24] proposed a cyber hierarchy and interactional network (CHAIN) framework utilizing an end–edge–cloud architecture for a cloud-based BMS. The CHAIN framework can provide multi-scale insights, and with that, more advanced and efficient algorithms can be developed for the state of charge and state of health estimation, thermal management, cell balancing, fault diagnosis, and other BMS functions. The proposed cloud-based BMS presented battery performance better visually, stored more battery data to help develop more accurate algorithms, and provided support for the development of optimal battery system control strategies.

Data mining and machine learning methods, which require large computation and memory, have been implemented in various fields, as the IoT systems and cloud platforms such as Amazon Web Services, Google Cloud Platform, and Microsoft Azure have become progressively more advanced, available, and affordable [61,62,63]. Support-vector machine [64,65], Gaussian process regression [66], neural networks [67,68,69], Markov chain [70], and fuzzy logic [71,72,73] are methods that have been used for battery state estimation and fault diagnosis, giving promising results, yet being considered impractical due to the limitations of the BMS. The cloud-based BMS will have high computational power, limitless data storage capability, and great system reliability. It can perform battery managing functions, like monitoring, diagnostics, prognostics, and optimization, more accurately and reliably. Some advantages of the cloud-based BMS are shown in Table 1. In terms of hardware, the cloud solution will reduce some components required for local computing resulting in smaller devices yet with much greater computing power from the cloud and unlimited data storage. In terms of software, the cloud-based BMS will be more efficient for control and optimization of the system, have better monitoring and data visualization, and perform more accurate and reliable battery prognostics and diagnostics. The development of the cloud-based smart BMS will potentially enable a new level of smart controls toward the next generation of energy storage technologies, paving the way for mass adoption of renewable energy globally.

**Figure 2:**
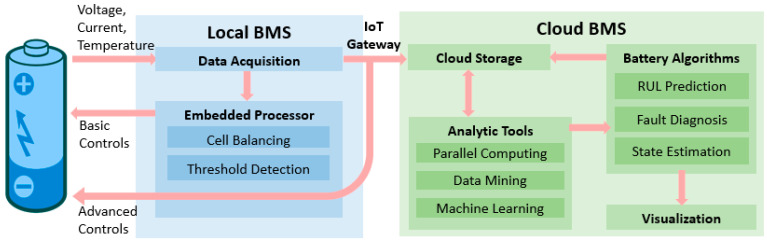
Potential design and functions of the cloud-based smart BMS.

The cloud-based smart BMS design is outlined in Figure 2. The hardware and software requirements for this type of BMS are more demanding than the current BMS design since it would need some extra components for cloud connection. The new design still has a local BMS consisting of the “slave” units. These “slave” units perform data acquisition by measuring the voltage, current, and temperature of battery cells in the pack using sensors at different sampling rates. There is also a “master” unit in the local BMS, but it would only perform basic safety functions such as cell balancing and fault threshold detection. These functions are better locally since they need to be performed fast in real time. 

As outlined in [22,23,24], extra components for cloud connection include an IoT component, a cloud infrastructure, an application programming interface (API), and a user interface (UI). For the IoT component, a fast and stable Internet connection like 5G is vital for real-time data transfer between the battery system and the cloud. As well, Raspberry Pi, which is a small single-board processing unit, can be utilized for data transfer and communication. The measured battery data can be sent to the IoT component by the “slave” units using the controller area network (CAN) protocol, and then forwarded to the cloud by the IoT component under the TCP/IP and message queuing telemetry transport (MQTT) protocol to ensure security and privacy. The cloud infrastructure usually consists of a data logger and a database. The data logger captures a large amount of non-structured or semi-structured data from the battery system and enables a secure gateway and data transfer into the cloud database. The database serves to store all the data to be used in the advanced battery data-analytic algorithms. The API, another component of the cloud-based BMS, acts as a bridge between the cloud database and the battery data analytics tools and algorithms, using popular programming applications such as Matlab and Python. The UI component of the BMS is necessary for the users to inspect and visualize the operation and state of the battery pack in real time, as well as investigate historical operation data which can help the users schedule maintenance and repair. This is a significant addition compared with the current BMS design which offers little data visualization. The users can be informed by the UI as soon as any battery system fault is identified through alarms, which help to reduce damaging effects to the batteries, ultimately improving the safety and reliability of the battery system and reducing maintenance costs.

## 4. Perspectives on the Functionality and Usability of Cloud-Based Smart BMS

The development of an efficient, reliable, and accurate BMS plays a very significant role in safe and effective battery management and control. The cloud-based smart BMS is a step in the right direction for future battery applications. However, there are still many points to address before it can become practical. For example, there should be a logical split of BMS features and functionalities to utilize both the local processing unit and the cloud platform effectively and efficiently. The functions of a smart BMS, mentioned in previous sections, include data acquisition, cell monitoring, cell balancing, charge control, SOC and SOH estimation, thermal management, fault detection, and fault prognosis. The risks and integration approaches for this type of BMS are other points that should also be brought up in discussion.

The local functions of the BMS should include data acquisition, cell balancing, charge control, thermal management, and fault detection. Some of these functions, however, can be supplemented by the use of the cloud platform. Data acquisition must be performed locally since it is done through physical sensors connected to the battery system. The data cannot be stored locally, therefore, it is transferred to the cloud and stored there to be used to perform other BMS functions. Cell balancing is required as the inconsistency and discrepancy among the cells may negatively affect the pack’s capacity, lifespan, and other important performance. Cell balancing for voltage can be done effectively with the local unit, and therefore does not have to utilize the cloud. However, cell balancing for SOC and capacity could benefit from data-driven equalization strategies which would require cloud computing and storage. Charge control prevents the battery system from overcharging and overdischarging and is another function that can be done effectively with the local BMS unit. Thermal management often involves cooling fluids or materials of which flow is controlled and adjusted based on the measured temperature, using control and feedback algorithms. Even though this function can be done locally, the use of the cloud can help improve it for future use, such as selecting a better cooling material or optimizing the control algorithms. Fault detection is another function that should stay in the local BMS units since it needs to be reliable and done in real time. The cloud platform depends largely on the Internet connection which may not be completely reliable at all times, and hence, it is better and inherently safer to have the fault detection function offline.

The cloud functions of the BMS should include but are not limited to, cell monitoring, SOC and SOH estimation, and fault prognosis. The cell monitoring function can be improved significantly with the help of the cloud platform, as it has better visualization, user interface, and more historical data, instead of a black-box solution such as the current offline BMS. As the current BMS has limited data storage, the cloud-based solution would essentially offer an unlimited amount of data storage. The estimation of SOC and SOH has always been a problem for the offline BMS, as it relies on the accuracy of the battery model and the estimation algorithms, which cannot be complex due to the computing limitations. With the cloud platform enabling high computing capability, users can develop and implement more accurate battery models (electrochemical models) and estimation algorithms (machine learning algorithms) for the SOC and SOH of battery systems. Fault prognosis is a supplementary function to the local fault detection function of the BMS. Instead of detecting faults as they occur, fault prognosis would use historical data and machine learning to predict where and when a fault might happen in the battery system, and notify the users to anticipate or prevent the fault. This function is also useful in terms of user behavior warnings as well as warranty tracking. There are other potential BMS functions that are not possible with the current offline BMS, such as lifetime prediction, economic optimization, etc., that can be developed with the implementation of cloud-based smart BMSs. Since this is a relatively new concept, there should be more discussions and research efforts going toward the efficient and effective split of the local and cloud functions for the smart BMS.

There exist some drawbacks associated with the cloud-based solution for the smart BMS. Firstly, a fast and reliable Internet connection is a mandatory component of the system. However, this might not be feasible at certain locations or for certain applications, such as mining or remote areas exploration. Moreover, even for common battery applications, Internet outages might affect the performance of the smart BMS. Backup power and Internet connection are thus necessary to ensure the smooth operation of the battery systems. Secondly, costs are also an important component of the cloud-based BMS that needs to be investigated more thoroughly. The costs of operating and maintaining the cloud platform depend largely on the amount of data and algorithms used on the cloud. Therefore, researchers need to identify useful battery data and optimal data collection frequency in order to minimize the costs of the cloud operation without sacrificing the accuracy and reliability of the BMS functions. Finally, since it is a relatively new concept for LIB systems, it needs to be implemented and tested in a small number of battery applications first, before it can be used widely in other applications. Applications that can benefit greatly from cloud-based smart BMS include EVs and large-scale BESSs due to the scale of data collection and the complexity of desired battery algorithms, and in these applications, a cloud solution implementation would also be easier and more sound.

## 5. Conclusions

With the rise of LIBs, BMSs play an important role in ensuring safety and optimizing the operation of battery applications. However, the current BMS design shows several critical drawbacks. Recent developments in cloud-based smart BMS design will address a major issue of the current BMS, which is the unreliability and inaccuracy of its battery algorithms due to limited computational capability and data storage. This study reviews the concept of the cloud-based BMS and its potential functionalities and usability. The cloud solution will provide a better solution for condition monitoring, fault prognosis, and optimization of LIB systems. The flexibility of the cloud system will also allow it to be used with minimal modifications in future ESS solutions such as solid-state LIBs, lithium–sulfur batteries, or metal–air batteries. By improving the reliability and performance of LIB systems, the cloud-based smart BMS will contribute to the mass adoption of renewable energy, making energy cheaper, cleaner, and more accessible to the public. With such benefits, there are still several drawbacks, such as the need for fast and reliable onsite Internet, prevention of outages, potentially higher yet unoptimized costs, and further proof-of-concept work and developments. Future research should focus on implementing advanced battery models and algorithms onto the cloud-based BMS and validating them in real-life battery applications.

## Figures and Tables

**Table 1:** Advantages of the cloud-based smart BMS.

BMS Components	Advantages
Hardware	Potentially smaller devices Significantly greater computational capability Virtually unlimited data storage
Software	More efficient operational control and optimization Better and more interactive monitoring and visualization More accurate and reliable prognostics and diagnostics
